# The Last Mile Problem: A Critical Assessment of Physics-Based and AI Tools for Small Molecule Binding Prediction in Virtual Screening

**DOI:** 10.1021/acs.jcim.6c00942

**Published:** 2026-05-07

**Authors:** Xiaowen Wang, Hamza Hentabli, Akhila Mettu, Shubha Gautam, Dmitri Kireev

**Affiliations:** Department of Chemistry, College of Arts and Sciences, 14716 University of Missouri, Columbia, Missouri 65211, United States

## Abstract

Docking-based virtual screening (VS) is essential for hit finding in the initial stage of drug or probe discovery. However, it remains prone to high false-positive rates, often resulting in unsuccessful screening campaigns. MD-based alchemical free-energy methods offer a promising solution to improve VS hit rates but are highly resource-intensive. Real-world and benchmark studies incorporating alchemical absolute binding free energy (ABFE) calculations could help optimize their use in VS pipelines. Here, we present a large-scale benchmark to evaluate the comparative value of ABFE calculations in VS workflows. Two data sets were used: a curated set of 632 ligand–protein complexes from the PDBbind database to assess ABFE quantitative accuracy and a set of 315 binders and decoys from the Database of Useful Decoys (DUD-E) to evaluate predictive power in a VS context. Alongside alchemical ABFE, we benchmarked computationally affordable end-state physics-based methods and five machine-learning (ML) models. The study ranked BFE predictors consistently with their computational cost, with alchemical ABFE performing well across both benchmarks. End-state methods scored well in recognizing actives from decoys in the DUD-E data set but showed little correlation with experimental values in PDBbind. Most ML models performed well on PDBbind, likely due to training overlap, but failed on DUD-E, except for GNINA and Boltz-2, which demonstrated a degree of generalization comparable to end-state physics-based methods. Overall, a staged approach involving Boltz-2 as a primary filter followed by alchemical ABFE is likely to robustly and cost-efficiently enrich docking-based VS hit lists with true actives.

## Graphical abstract

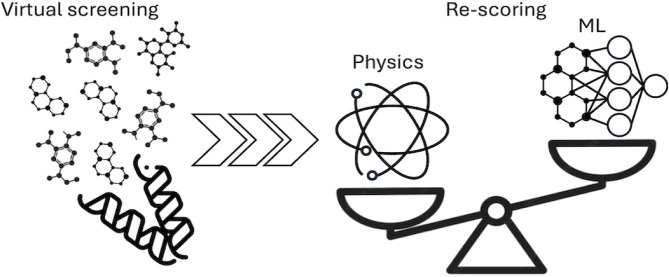

## Introduction

Over the years, docking-based virtual screening (VS)^1,2^ for identifying hit molecules to initiate drug discovery projects has gone mainstream. Moreover, VS has proven indispensable for small pharmaceutical companies and academic laboratories lacking access to extensive legacy compound collections. Unlike experimental screening methods, VS does not require compounds to be physically available; only a small subset of computationally prioritized hits needs to be procured for experimental validation. Numerous docking algorithms^3–5^ are now publicly available and have demonstrated value in terms of both performance and binding pose accuracy. More recently, open-source ML-powered methods have expanded the pool of docking tools, offering tangible improvements.^6–8^ Despite these advancements, in silico screening is still prone to high false-positive rates, which can result in unsuccessful campaigns—an experience many virtual screeners have likely encountered. Until recently, the only quantitative estimates of virtual screening efficiency in the public domain were published case studies. According to comprehensive reviews, the experimentally confirmed hit rates frequently exceed 20%.^9,10^ However, these estimates are highly likely to be skewed by survivorship bias, which refers to the reality that unsuccessful screening campaigns are almost never published. Consequently, the apparent rate of success in the public domain is artificially inflated because the community only has access to the outcomes of highly successful virtual screening campaigns on targets with prior knowledge of ligands. To gain a more objective understanding of VS efficiency, the Critical Assessment of Computational Hit-Finding Experiments (CACHE) Challenges were initiated in 2021.^10^ Participants in each challenge are tasked with performing VS against a specific target and submitting a list of 100 virtual hits for procurement and experimental validation. Results from CACHE Challenge #1 showed that only 7 of 23 participants identified any experimentally confirmed hits,^11^ and in CACHE Challenge #2, this number was only 5 out of 25.^12^ What factors contributed to the success of the winning strategies? Interestingly, it was not the choice of docking algorithm, as most participants, including the winners, relied on widely used software such as AutoDock-GPU,^13^ AutoDock Vina,^14^ or Glide.^15^ Instead, success appeared to stem from other aspects of the VS workflow, such as targeted preselection of ligands prior to docking,^16^ enhanced scoring functions,^6^ or the extensive use of MD-based absolute and relative binding free energy (ABFE/RBFE) calculations.^17^ MD-based alchemical free energy methods are often considered the ultimate solution to increasing VS hit rates, potentially enabling the identification of true actives in a small set of experimentally tested hits.^18,19^

Alchemical free energy calculations provide a rigorous, physics-based framework for estimating binding affinities.^19–22^ Alchemical methods assess ABFE through a process of slowly morphing the protein-bound ligand into nothing. Typically, this ligand annihilation proceeds through discrete, chemically impossible (aka alchemical) steps called λ-windows. The free energies between consecutive alchemical states can be calculated through a statistical analysis of the system’s energy differences between respective states for all sampled configurations. These alchemical energies are then summed to obtain the final ABFE value. The high similarity between states in the same λ-window ensures that both states have equivalent configuration spaces, a prerequisite for the applicability of the approach. These calculations are not perfectly accurate, though. The sources of error include the molecular mechanics force field (which is only an approximation of true quantum interactions), insufficiently fine windows, and undersampling of configuration spaces. Moreover, these calculations are computationally expensive, requiring significant time and resources. For a typical VS study, a laboratory could readily afford ABFE calculations for up to 100 compounds. Yet, there is a substantial risk that a list of 100 docking hits may not contain any actives at all, and the entire list would need to be experimentally tested for reliable results. To fully leverage ABFE calculations, they must be applied to a much larger set—up to 1,000 molecules—to ensure an enriched hit list of fewer than 100 compounds for experimental testing. Even as computing resources continue to become more powerful and less expensive, the cost of running alchemical simulations for hundreds of ligand–protein complexes is likely to remain significant for the foreseeable future. Would such a resource-intensive effort yield the desired outcomes? While the theory underpinning alchemical calculations is thorough, practical applications may be hindered by imperfect force fields or an insufficient number of λ-windows. Additionally, incorrect docking poses for certain ligands can lead to erroneous ABFE predictions. To date, there is no clear consensus on the optimal application of resource-intensive methods for securing an enriched VS hit list for experimental confirmation. Moreover, the costs per compound in this final step are orders of magnitude higher than in all previous steps, leaving little room for waste—a challenge commonly referred to in delivery and telecommunication logistics as “the last-mile problem”. Real-world and benchmark studies incorporating ABFE calculations could facilitate their effective use in VS pipelines.

Toward this goal, we present a large-scale benchmark study, which, to the best of our knowledge, is the first of its kind, to evaluate the comparative value of ABFE calculations in VS workflows. In this study, we first established an accuracy benchmark by performing affinity predictions for a curated set of 632 ligand–protein complexes (comprising 206 unique proteins and 606 unique ligands) with experimentally determined binding affinities (*K*_d_/*K*_i_ values) and 3D structures from the PDBbind database.^23^ Second, we evaluated affinities for a set of 135 true binders and 180 decoys selected from the Database of Useful Decoys–Enhanced (DUD-E).^24^ A hypergeometric probability distribution function^25^ was applied to estimate the enrichment that would have been achieved by each benchmarked technique in a real-world VS study. Overall, we evaluated three physics-based methods: (i) alchemical ABFE,^22,26^ (ii) Molecular Mechanics/Poisson–Boltzmann Surface Area (MM/PBSA),^27^ and (iii) MM/Generalized Born Surface Area (MM/GBSA)^27^—alongside seven machine learning (ML) models: GNINA,^6^ K_DEEP_,^28^ OnionNet-2,^29^ TopologyNet,^30^ Yuel,^31^ RF-score-VS,^32^ and Boltz-2.^33^ MM/PBSA and MM/GBSA are end-state methods, requiring MD simulations only for ligand–protein complexes and unbound ligands. While end-state methods require similar computational time per affinity calculation as alchemical ABFE techniques (on the order of hours), they utilize an order of magnitude fewer compute nodes, enabling significantly higher parallelization. Thus, it is essential to assess these less resource-intensive alternatives. Finally, we included ML-based affinity predictors as an even more affordable option. Since the advent of deep neural networks,^34^ ML models have shown promise in affinity prediction. The five ML models were selected based on the diversity of their underlying architectures, reported performance, and availability of source code.

## Results

### Data Sets

Two data sets were used in this study. One of them, the *PDBbind benchmark set*, is composed of ligand–protein complexes with experimentally determined affinity values and X-ray structures. This is a reference data set that allows us to assess the accuracy of the benchmarked methods in quantitatively predicting ligand–protein binding free energy for known binders. The second one, the *DUD-E benchmark set*, is used to assess the ability of the methods to distinguish between binders and nonbinders using docking poses as starting points for MD simulations and ML methods that require 3D input information. The performance of the benchmarked methods on this second data set would be indicative of how useful they might be in a VS setting.

The primary requirement for the reference affinity data set was the availability of experimentally determined 3D structures and affinity data expressed as *K_d_*/*K_i_*. The refined PDBbind subset contains 5,316 protein–ligand complexes formed by 4,131 unique ligands and 1,409 unique proteins.^35^ Since the ultimate goal of this study is to evaluate the potential of BFE predictors in hit finding, we focused exclusively on ligands that could potentially serve as leads in drug discovery projects. To this end, we retained ligands that satisfy Lipinski’s rules,^36^ with the exception of molecular weight, which we restricted to the range of 250–600 Da. We also excluded ligands with more than one ionizable group, those containing phosphorus atoms, and those featuring potentially toxic or highly reactive groups (see Methods for details)—these filters were implemented to ensure that the benchmarked compounds possess a physical and chemical profile similar to molecules that might potentially be considered viable leads in drug discovery projects. Compounds with multiple ionizable groups or phosphorus atoms frequently exhibit poor pharmacokinetic properties, such as low membrane permeability, making them unrepresentative of desirable starting points for lead optimization. Additionally, we excluded complexes involving metalloproteins, as standard force fields do not accurately represent metal coordination interactions.^37–39^ Eventually, a data set of 632 protein–ligand complexes involving 206 unique proteins and 606 unique ligands was retained for all benchmark calculations (see Supplementary Figure S1 for probability density functions (PDF) of the key physical properties for the data set compounds, Supplementary Data set 1 for a compressed set of PDB files of the protein structures, and Supplementary Data set 2 for unique ligand structures in SMILES format). The protein structures belong to 124 protein structure superfamilies (as defined in the SUPFAM database^40^), such as protein kinase-like or methyltransferase (see Supplementary Table S1 for the protein family list). Each protein is associated with 1 to 56 ligands (see Supplementary Data set 3 for detailed information on ligand counts per target), and nine targets are associated with more than ten ligands. Targets with high ligand counts are particularly useful for determining whether binding free energies can be more accurately predicted for individual targets compared to the entire data set. Finally, we estimated the chemical diversity by the Butina clustering algorithm, with the inclusion criterion for the Tanimoto coefficient set to 0.5 (on ECFP4 fingerprints). A total of 379 clusters were obtained, with cluster sizes ranging from 1 (263 singletons) to 10 (there are five clusters with more than 5 compounds).

The binders/nonbinders data set was selected from the DUD–E database.^24,41^ Here, the rationale was to test the benchmarked methods under conditions close to those in virtual screening campaigns. Hence, statistically significant numbers of known binders and decoys need to be evaluated against a single target (here, “decoys” means nonbinders having physical properties similar to those of binders). To satisfy the latter requirement while keeping the overall number of compounds affordable for alchemical ABFE calculations, the number of targets must be significantly smaller compared to the PDBbind data set. Previously, we created a DUD-E subset involving 10 structurally diverse targets broadly covering the protein space.^42^ Nine of these targets—ACE, ADRB1, FAK1, GRIK1, HMDH, MCR, PGH2, PRGR, and TRYB1—are also present in the reference PDBbind data set and cover the most important drug classes, including kinases, proteases, GPCRs, nuclear receptors, ion channels, and synthases. This data set features a total of 315 ligand complexes—135 binders (15 per target) and 180 decoys (20 per target). Importantly, experimentally determined 3D structures are not available for most of the ligand–protein complexes in this DUD-E subset, as well as quantitative affinity data, which is another distinctive feature making it more relevant to a docking-based VS setting. The AutoDock Vina method was used to produce docking poses in this study.^14^ A compressed set of PDB files with docking-based ligand–protein complexes for the DUD-E data set is made available as Supplementary Data set 4 and the unique ligand structures in SMILES format as Supplementary Data set 5.

### PDBbind Benchmark

#### Alchemical ABFE Calculations

We first establish a reference benchmark by calculating BFE for the PDBbind data set using the theoretically rigorous alchemical ABFE calculations. We used a standard ABFE protocol as implemented in GROMACS.^43,44^ Free energy differences between the alchemical states were estimated using the BAR method.^45^ The key parameters in an alchemical ABFE protocol are the number of λ-windows and the size of each λ-window. The number of λ-windows was set to 25, a frequently observed choice across recent publications.^21,46–48^ The length of a λ-window was determined by running ABFE simulations with window sizes of 1 ns, 5 ns, 10 ns, and 15 ns on a subset of 77 ligand–protein complexes with uniformly distributed experimental binding affinities randomly pulled from the PDBbind data set. Since the computational cost of an alchemical calculation is directly proportional to the λ-window length, it is important to identify the length corresponding to an optimal cost-accuracy balance. Expectedly, ABFE calculations with longer λ-windows tend to be more accurate based on Pearson’s correlation coefficient (*r*) and Spearman’s rank correlation coefficient (ρ) between the calculated and experimental BFE values and the respective scatter plots (Figure 1A–D). However, the gain in accuracy for 10 ns and 15 ns windows (over the 5 ns window) is relatively modest given the additional computing power that such calculations would require. Therefore, the 5 ns λ-window was used throughout the study.

**Figure 1:**
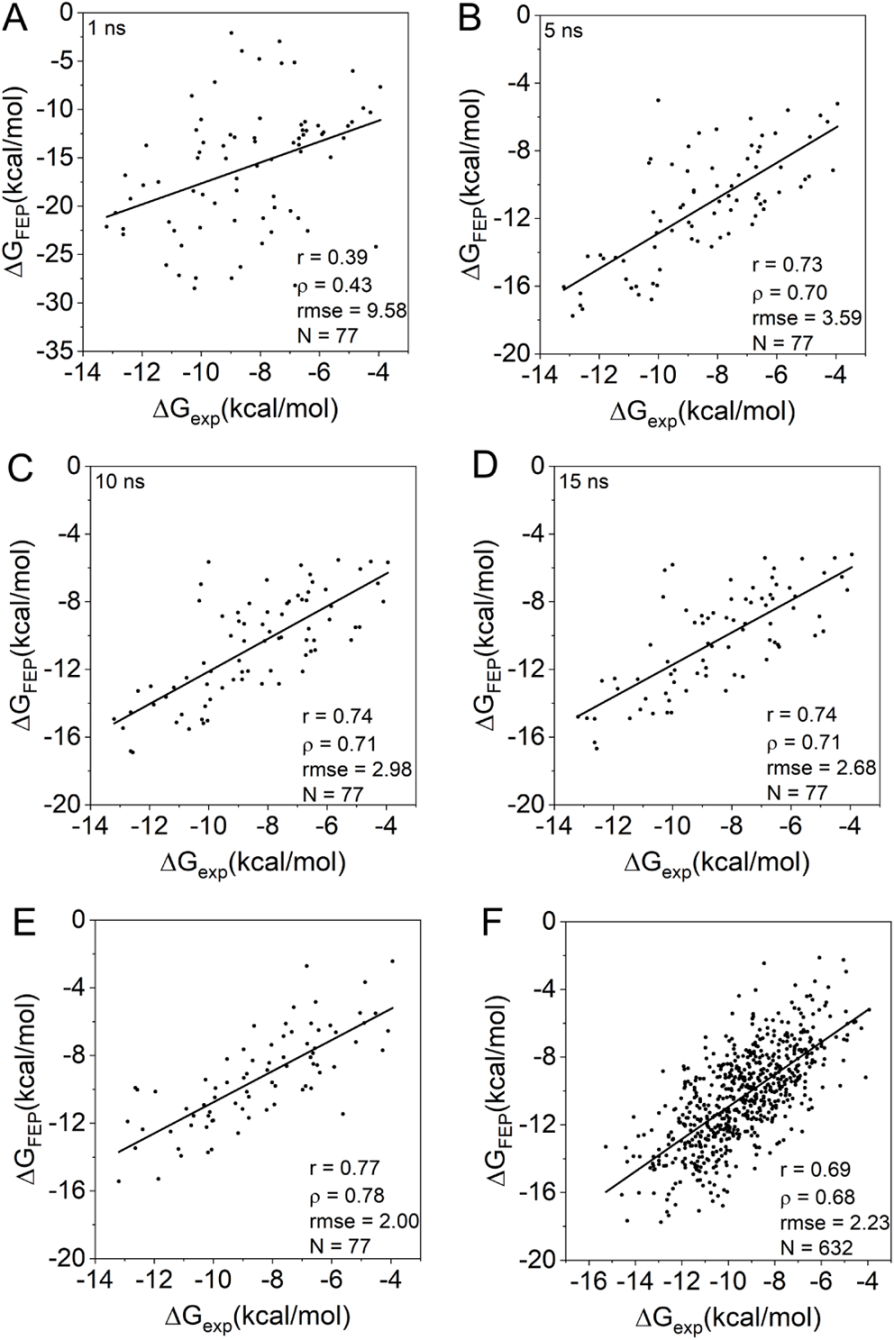
Correlation between alchemical (Δ**G_FEP_**) and experimental (Δ**G**_exp_) BFE on the PDBbind data set. Simulations with λ-window lengths of 1, 5, 10, and 15 ns, respectively, on a pilot subset (A–D); with orientational restraints setting (E), and for the full data set (F).

We also examined the effect of applying spatial restraints that maintain the nearly decoupled ligand close to its initial binding pose. Such restraints are often recommended to improve thermodynamic reversibility. Therefore, it was of interest to assess whether they are truly necessary in our simulation context. To this end, we performed additional ABFE calculations with orientational restraints for the same 77 complexes used in the window size optimization (Figure 1E). While the application of restraints resulted in improved correlation metrics (with Pearson r increased from 0.73 to 0.77), this improvement must be weighed against practical trade-offs in the context of VS triage. In particular, implementing spatial restraints requires additional λ-windows, which increases the computational cost by up to 30%. Furthermore, automating the assignment of these restraints across highly diverse ligands and binding pockets introduces significant complexity and potential points of failure in a computing pipeline. Therefore, because the unrestrained setup still achieved a highly respectable correlation of r = 0.73, we believe that it provides an optimal balance of accuracy, computational speed, and ease of automation. These practical considerations, alongside previous studies showing that comparable accuracy can often be achieved without restraints^20,49^ motivated our decision to use the unrestrained setup for the full data set.

A total of 632 simulations were run for all complexes of the PDBbind data set. The scatter plot of calculated vs experimental BFE for all PDBbind complexes is shown in Figure 1F (all calculated and experimental BFE values are available as Supplementary Data set 6). The Pearson’s *r* = 0.69 and Spearman’s ρ = 0.68 for the whole data set are slightly lower than those obtained on the pilot subset of 77 complexes (*r* = 0.73 and ρ = 0.70) (Figure 1B). We sought to understand the reasons for this decline and, more generally, how the BFE calculation accuracy may vary with properties of proteins or ligands. Such an analysis may help to anticipate how successful the application of alchemical ABFE calculations might be in a particular VS project. First, we examined intratarget correlations—defined as correlations calculated exclusively among sets of different ligands binding to the same protein—for targets featuring at least 10 ligands. We found that the exp./calc. correlations vary across proteins, with Pearson’s *r* ranging from 0.44 to 0.75 and Spearman’s ρ ranging from 0.19 to 0.74, suggesting a dependence of the calculation accuracy on the nature of protein–ligand complexes (Supplementary Figure S2, Supplementary Table S2). Several plausible factors that could affect the accuracy of ABFE calculations—and be accounted for prospectively—include the size of the protein or ligand (measured by molecular weight), the fraction of the ligand immersed in the protein (approximated as the median number of nearby protein atoms per ligand atom), and the binding pocket composition (expressed as the mean clogP of the pocket residues). We tested whether ABFE calculation accuracy differed for complexes with the above factors above and below their median values. However, the ABFE values for all of the split subsets show similar levels of correlation with the experimental data and hence cannot be used as useful criteria to prospectively assess the accuracy of ABFE predictions (Supplementary Figure S3).

Additionally, we investigated whether the accuracy of the alchemical approach depends on the ligands’ affinity. To this end, we calculated the root-mean-square error (RMSE) for the top 50% of binders (RMSE = 2.30 kcal/mol) and the bottom 50% of binders (RMSE = 2.16 kcal/mol). The difference between these two subsets is insignificant, indicating that tighter binders are not simulated more accurately than weaker binders.

#### End-State Methods

In addition to theoretically rigorous alchemical calculations, we explored the value of two popular empirical simulation-based methods. Both MM/PBSA^27^ and MM/GBSA^27^ are end-state techniques; that is, MD simulations and subsequent analyses involve only bound and fully decoupled ligands and proteins. MD trajectories of 5 ns were used for both MM/PBSA and MM/GBSA. The end-state calculations were performed in three modes: (i) without the entropic correction, (ii) with entropic correction by normal-mode analysis (NMA),^50,51^ and (iii) with entropic correction by interaction entropy (IE),^52^ which is more computationally affordable than NMA. The outcome of both end-state methods, with and without entropic corrections, is summarized in Figure 2A–F (all calculated values are available as part of Supporting Information Data Set 6). Overall, none of the methods demonstrated satisfactory performance, and, consistent with earlier evidence,^53^ the entropy-corrected methods performed even worse than the original MM/PBSA and MM/GBSA.

**Figure 2:**
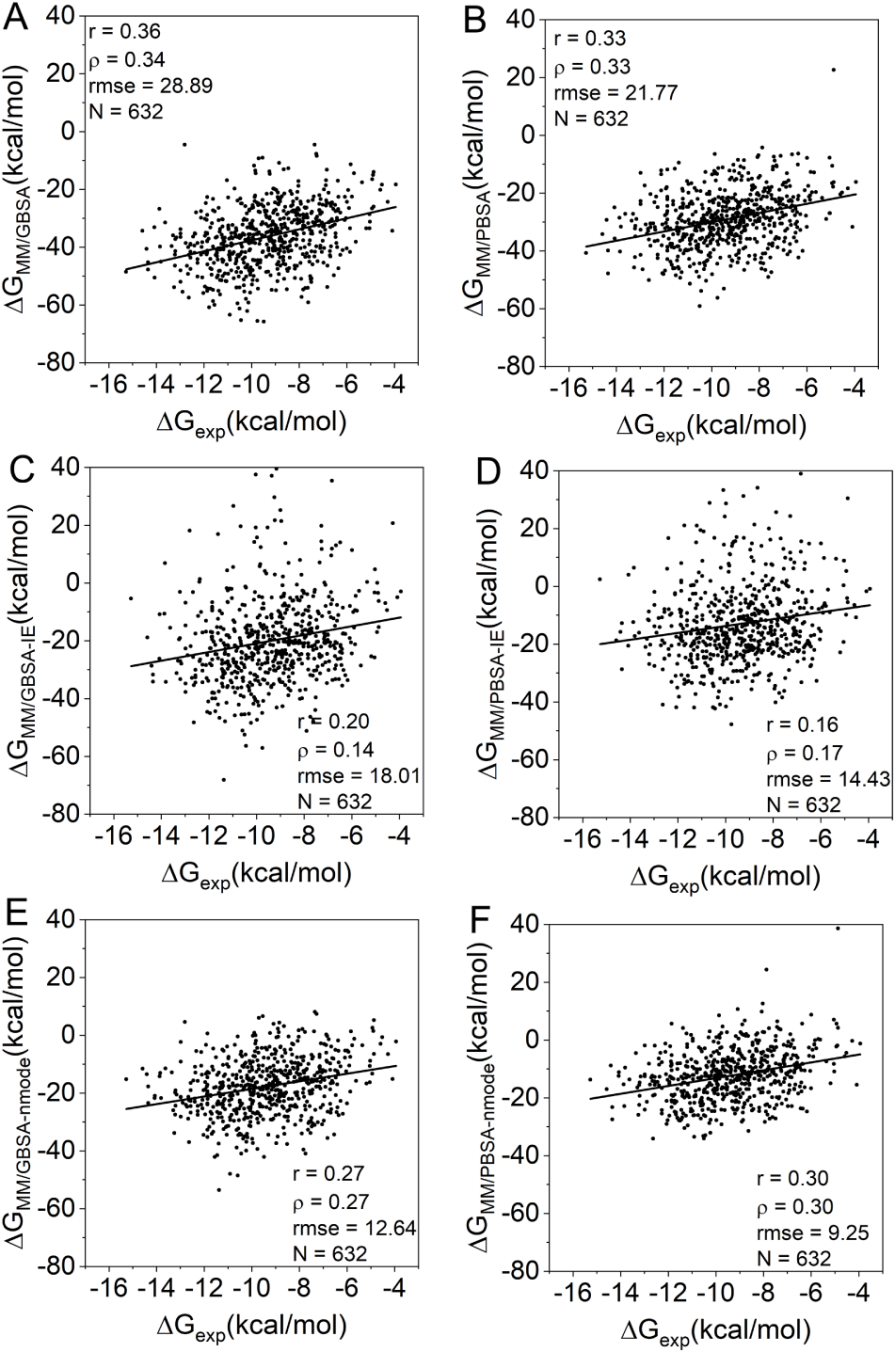
Correlation between experimental BFE (Δ**G_exp_**) and the end-state methods: (A) MM/GBSA (ΔG_MM/GBSA_), (B) MM/PBSA (ΔG_MM/GBSA_), (C) MM/GBSA with IE correction (ΔG_MM*/*GBSA–IE_), (D) MM/PBSA with IE correction (ΔG_MM/PBSA–IE_), (E) MM/GBSA with normal mode correction (ΔG_MM/GBSA–nmode_), and (F) MM/PBSA with normal mode correction (ΔG_MM/PBSA–nmode_).

Intratarget correlations, with a few notable exceptions, align with those observed in the full PDBbind data set (Supplementary Table S2). The MM/GBSA data showed a Pearson’s *r* greater than 0.5 for only two targets (BRD4 and WDR5), while the MM/PBSA data identified three such targets: BRD4, MK14, and WDR5.

In addition, we examined whether the accuracy of the end-state methods depends on specific properties of the ligand–protein complexes, including protein and ligand molecular weights, the fraction of the ligand buried in the protein, and binding pocket lipophilicity. The accuracy of both end-state methods appeared unaffected by any of these factors, as illustrated in Supplementary Figures S4 and S5.

#### ML Models

The seven ML models were selected to represent the diversity of approaches developed since the revival of interest in applying machine learning to chemistry. For instance, KDEEP^28^ makes use of a 3D-convolutional neural network (CNN) trained on 3D structures of ligand–protein complexes; chemical structures are described as pharmacophoric features. OnionNet-2 ^29^ is a graph-CNN; the input graph nodes are ligand atoms and whole protein residues. TopologyNet^30^ is a 1D CNN model in which ligands and proteins are represented by topological fingerprints. Yuel^31^ is a combination of a CNN (for proteins) and a graph-CNN (for ligands), which are plugged into a multilayer perceptron (MLP); input ligands are represented as SMILES, and proteins as sequences. RF-Score-VS^32^ is a random forest model that relies on the occurrences of intermolecular atom–atom contacts to predict binding affinity. Most of the above methods are trained and tested on subsets of the PDBbind database.^23^ Finally, GNINA^6^ and Boltz-2 ^33^ somewhat differ from the other methods: GNINA’s affinity model (combining CNN and a physics-based function) is embedded in the docking process, so that the final ligand pose is selected to minimize the predicted BFE value, and Boltz-2 is a cofolding model that predicts binding affinities along with protein–ligand complex structures. The results of applying the seven ML methods to predict BFE values for the 632 PDBbind set are summarized in Figure 3 (all calculated values are available as part of Supplementary Data set 6). Two methods, K_DEEP_ and OnionNet-2, demonstrate accuracy comparable to that reported in their respective original publications. In contrast, two other methods, TopologyNet and Yuel, show markedly lower performance, with a Pearson correlation slightly exceeding 0.5 compared to approximately 0.75 as reported in the original sources. Boltz-2 achieved a similar intermediate performance, yielding both a Pearson r and Spearman ρ of 0.58. Finally, GNINA and RF-Score-VS showed the weakest performance in terms of correlation with experimental data. GNINA produced a Pearson r = 0.34 (Spearman ρ = 0.52), while RF-Score-VS yielded a Pearson r = 0.30 (Spearman ρ = 0.35). The intratarget correlations are consistent with the general trends (see Supplementary Table S2 for details).

**Figure 3:**
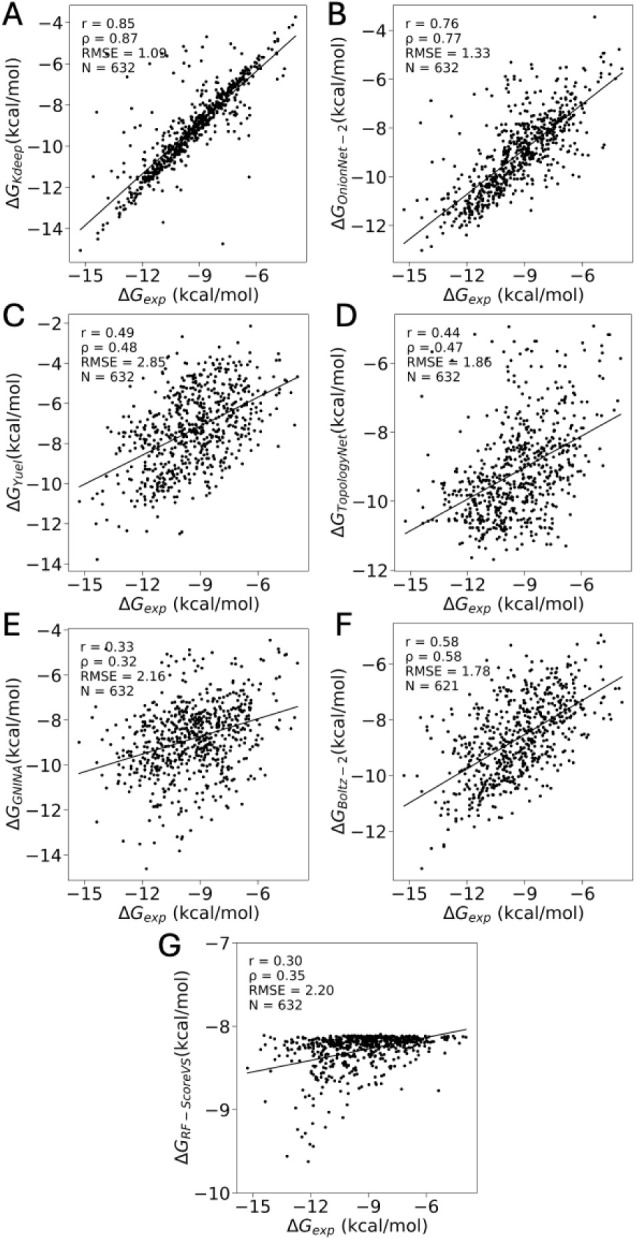
Correlation between experimental BFE (ΔG_exp_) and the ML models: (A)­K_DEEP_ (ΔG_KDEEP_), (B) OnionNet-2 (ΔG_OnionNet–2_), (C) Yuel (ΔG_Yuel_), (D) TopologyNet (ΔG_TopologyNet_), (E) GNINA (ΔG_Gnina_), (F) Boltz-2, and (G) RF-ScoreVS.

### DUD-E Benchmark

#### Performance Metrics

After establishing an accuracy reference on a set of ligand–protein pairs with known 3D structures and BFE values, we assessed how well the benchmarked methods discriminate between binders and decoys from the DUD-E data set. Quantitative metrics such as sensitivity, specificity, and ROC-AUC^54,55^ can be calculated by using standard classification counts. However, the small size of our benchmark data sets (15 actives and 20 decoys per target) and the artificially high fraction of active ligands make these standard metrics less representative of a real-world virtual screening setting. In a practical scenario, a laboratory might typically select 100 ligands for experimental testing from a larger docking-generated hit list. Based on hit rates reported in CACHE Challenges 1 and 2,^12,56^ the true positive rate of a primary docking screen is approximately 1%. Therefore, identifying true actives from a 100-compound selection typically requires rescoring at least 1,000 docking hits, which would contain approximately 10 true actives.

To make our performance metrics interpretable in this practical context, we employed the hypergeometric distribution for two specific purposes. First, we used it to establish a quantitative statistical estimate of how the observed true positive rates compare to random compound selection. It provides the exact probability, *P­(k)*, of successfully identifying *k* true positives when randomly selecting *n* compounds from a total set of *N*_*tot*_ ligands that contains exactly *N*_*pos*_ active compounds:

1 $$P\left(k\right)=\frac{\left(\begin{array}{l} N_{pos}\\ k \end{array}\right)\left(\begin{array}{l} N_{tot}-N_{pos}\\ n-k \end{array}\right)}{\left(\begin{array}{l} N_{tot}\\ n \end{array}\right)}$$

In our benchmark setup, *N*_*tot*_ = 35 and *N*_*pos*_ = 15 per target, and we selected *n* = 7 ligands to evaluate the success rate. Methods that provide significant predictive value show true-positive rates that are virtually impossible to observe by random chance under this exact distribution. Second, we used the hypergeometric distribution to mathematically project these benchmark results onto the more realistic virtual screening scenario of *N*_*tot*_ = 1 000, *N*_*pos*_ = 10, and *n* = 100. By matching the cumulative probabilities between our specific benchmark setup and this larger hypothetical distribution, we translated the raw performance on our limited data set into a meaningful estimate of how each method would enrich the hit rate in a practical, large-scale screening campaign where active compounds are exceedingly rare (see Methods for details).

#### Alchemical ABFE Calculations

Overall, the probability density functions (PDFs) of BFE values for binders and decoys (Figure 4) show clearly distinct distributions, though with a certain overlap (all underlying data are available as part of Supplementary Data set 7). In a practical VS setting, where the number of decoys significantly outweighs the number of actives, this overlap may result in a higher false positive rate than observed in our benchmark study. In this benchmark, 59 of the predicted positives were true positives, corresponding to a TPR of 94%. The probability of observing this TPR in a randomly selected ligand list is near zero, (*P*(*k*)≈) 10^–20^, as calculated by eq 1. Extrapolating these statistics to a practical setting (*N*_*tot*_ = 1,000, *N*_*pos*_ = 10, and *n* = 100) by mapping the respective hypergeometric distributions suggests that all 10 actives would be recovered. Moreover, designating as few as 17 positives would still capture all 10 actives. This trend is consistent across all nine targets (see Supplementary Table S3), with a 100% TPR for five targets and 86% for the remaining four.

**Figure 4:**
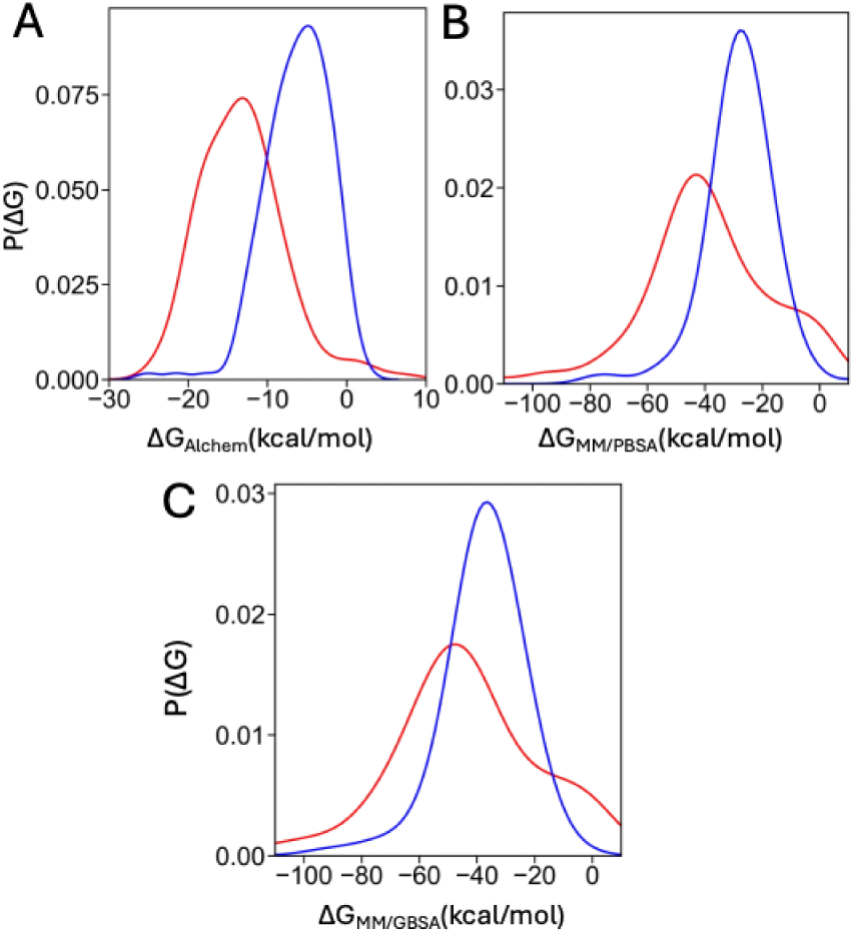
Probability density functions of calculated BFE values for actives (red) and decoys (black): (A) alchemical ABFE; (B) MM/GBSA; and (C) MM/PBSA.

#### End-State Methods

The PDFs of binding free energy (BFE) values for actives and decoys calculated using MM/GBSA and MM/PBSA (Figure 4) demonstrate a clear separation (all underlying data are provided in Supplementary Data set 7). The overall TPRs for MM/GBSA and MM/PBSA are 70% and 83%, respectively. The probability of observing these TPR values by random selection (calculated using eq 1) is extremely low (*P*(*k*) < 10^–6^).

Extrapolating MM/GBSA performance to a practical VS setting (*N*_*tot*_ = 1,000, *N*_*pos*_ = 10, and *n* = 100) suggests that 8 actives would be recovered. For MM/PBSA, all 10 actives would be recovered in a hit list of 100 ligands. Notably, a hit list of only 38 ligands would still recover all 10 actives with MM/PBSA. However, this overall high performance is not consistent across the nine targets (Supporting Information Table S3). For MM/GBSA, four targets produced hit lists with a TPR of 57%, which is comparable to a 43% probability of random selection. For one target, HMG-CoA reductase (HMDH), MM/GBSA performs significantly worse than a random selector, with a TPR of only 14%. The high overall MM/GBSA performance is largely driven by its strong performance on four targets: FAK1 (TPR = 100%), MCR (TPR = 86%), PRGR (TPR = 100%), and TRYB1 (TPR = 100%). In contrast, MM/PBSA demonstrates better performance consistency across the nine targets (Supporting Information Table S3). It underperforms significantly on only one target, HMDH, with a TPR of 29%, which is equivalent to that of a random selector. For the remaining targets, MM/PBSA achieved TPRs ranging between 71% and 100%.

The superior performance of MM/PBSA over MM/GBSA aligns with theoretical expectations.^27^ MM/PBSA numerically solves the Poisson–Boltzmann equation to provide a more rigorous physical representation of the electrostatic environment. In contrast, MM/GBSA relies on the generalized Born approximation, which is computationally faster but simplifies the treatment of polar solvation free energy and, therefore, often yields less accurate binding affinity predictions.

### Sensitivity to Starting Coordinates

Because experimentally determined X-ray structures were available for 55 of the 135 active ligand-protein complexes, we performed a retrospective analysis on how the source of the structural data affects the performance of physics-based methods. Of these 55 ligands, 43 yielded docking poses that closely resembled those of their corresponding X-ray structures. For the remaining 12 ligands, the docking poses deviated more significantly, exhibiting a root-mean-square deviation greater than 3 Å from the experimental coordinates. Using these docking poses as starting geometries for molecular dynamics instead of the X-ray structures resulted in a small overall performance improvement for MM/GBSA and a marginal decline for the alchemical method, whereas the performance of MM/PBSA remained unchanged. Specifically, for MM/GBSA, the performance on two targets (ADRB1 and GRIK1) improved from a random-like baseline (NTP = 4) to a fair enrichment (NTP = 5). Conversely, the alchemical method identified one fewer hit for PGH2 (NTP of 5 instead of 6). Of the 12 alchemical calculations started from divergent docking poses, five showed significant differences (greater than 2 kcal/mol) when compared to those initiated from X-ray structures. Four of these five calculations resulted in weaker predicted affinities, thereby increasing the risk of false negatives. This behavior is expected, as the native experimental pose should correspond to the lowest binding free energy. Nevertheless, our results demonstrate that even these underestimated affinities may still be sufficient for correct classification, resulting in no significant impact on the overall true positive rate (see Supporting Information Data Set 7 for BFE values on divergent X-ray/docking poses).

### ML Models

Visually, the probability density functions (PDFs) of binding free energy (BFE) values for actives and decoys calculated by five of the seven ML models (Figure 5A–E) seem to almost fully overlap (all underlying data are provided in Supplementary Data set 7). For these five earlier models, the overall TPRs vary between 20% and 38%. The probability of observing TopologyNet’s TPR of 20% in a random selection is 11%, which is the highest possible for the hypergeometric distribution with *N*_*tot*_ = 307, *N*_*pos*_ = 135, and *n* = 63. The event of observing K_DEEP_‘s TPR of 24% in a random selection is high as well (approximately 2%). Among these earlier methods, only GNINA, with an overall TPR of 38%, significantly differs from a random selector (*P­(k)* = 9.0 × 10^–04^). In stark contrast, the two more recently developed models, Boltz-2 and RF-Score-VS, achieved exceptional overall TPRs of 81% and 79%, respectively, with virtually zero probability of occurring by random selection (*P­(k)* = 2.1 × 10^–11^ and 1.6 × 10^–10^).

**Figure 5:**
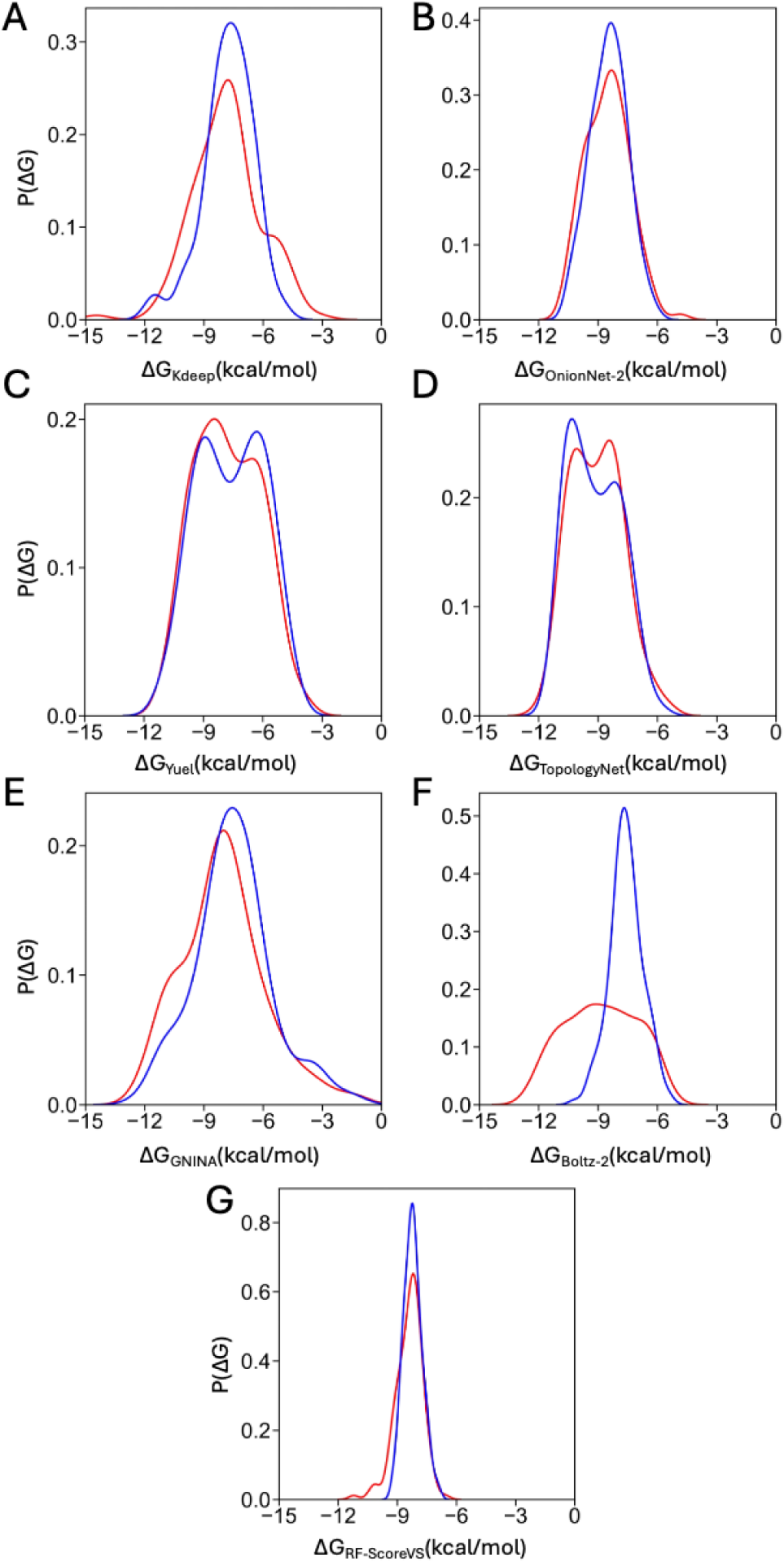
PDF of calculated BFE values for actives (red), decoys (black), and PDBbind (blue): (A) K_DEEP_; (B) OnionNet-2; (C) Yuel; (D) TopologyNet; (E) GNINA; (F) Boltz-2; and (G) RF-Score-VS.

Correspondingly, in a practical VS setting (*N*_*tot*_ = 1,000, *N*_*pos*_ = 10, and *n* = 100), four of the earlier ML methods (K_DEEP_, Yuel, OnionNet-2, and TopologyNet) would only be able to recover between 1 and 3 actives in a hit list of 100 ligands, representing an enrichment comparable to that of a random selection. They perform consistently poorly across the nine targets (Supplementary Table S4), with each method generating only three hit lists enriched better than a random selection. GNINA recovers 5 out of 10 hits in a practical VS setting, standing out among the other earlier ML methods. However, due to its poor performance on five out of nine targets, relying on it as even a preliminary filter in a practical VS study would be risky. Conversely, extrapolating the performance of the more recent Boltz-2 and RF-ScoreVS models to the practical VS setting suggests that both would successfully recover all 10 actives. Furthermore, unlike the earlier ML methods, Boltz-2 and RF-ScoreVS demonstrate strong performance consistency across the nine targets, both significantly underperforming on only two targets—HMDH and PGH2 for Boltz-2, and GRIK1 and HMDH for RF-Score-VS.

## Discussion

This work addresses a gap in the critical assessment of MD-based methods for virtual screening. In addition, we compare computationally intensive, physics-based approaches with ML techniques within a standardized benchmark protocol. While the application of alchemical and end-state methods in VS contexts has been previously discussed,^18,19,57^ no large-scale evaluation studies have been reported to date. Prior large-scale efforts exploring the potential of alchemical methods have primarily focused on relative binding free energy (RBFE) calculations to support lead optimization within chemical series;^49,58–60^ in these studies,^58–60^ the commercial software—FEP+^61^—was employed. ABFE evaluations, in contrast, have so far been limited to smaller-scale studies, as summarized in recent reviews.^62,63^ Only one of those studies specifically tested the performance of the alchemical ABFE approach in a VS-like setting, using small sets of actives and decoys against three targets.^64^ However, the reported ROC-AUC values— derived from data sets containing equal numbers of actives and decoys—do not accurately reflect performance in real-world VS campaigns. Interestingly, this study also found no correlation between experimental BFE values and calculated ABFE, potentially due to the use of short λ-windows (1–3 ns).

We designed our study to address several key questions: How well do alchemical ABFE calculations correlate with experimental BFE for complexes involving diverse protein targets and ligands? How effectively can alchemical ABFE distinguish binders from nonbinders? What is its estimated performance in a practical VS setting, where nonbinders greatly outnumber binders? How does it compare to more affordable BFE prediction methods, including physics-based approaches and ML models? To ensure accessibility and reproducibility, we used publicly available software (GROMACS^43,44^ ) and a standard ABFE protocol^65^ rather than the commercial FEP+ used in most previous large-scale studies.^58–60^ Although ABFE calculations remain an area of active research,^62,63,66,67^ we deliberately opted for a standardized implementation that could be readily adopted by nonexperts. Specifically, we determined a set of parameters that perform well across a broad range of protein targets. While some computational resources might have been saved by adopting variable-length λ-windows,^67^ we prioritized a more conservative protocol to enable automated, curation-free execution.

The choice of a benchmark data set is a critical factor in any retrospective virtual screening evaluation. In particular, the DUD-E data set we used for the VS-like benchmark has known limitations and biases, such as decoys that can sometimes be distinguished from actives based on simple physical properties rather than true binding interactions, and limited chemical diversity among active compounds for given targets.^68,69^ While newer data sets such as MUV,^70^ DEKOIS 2.0,^71^ and LIT-PCBA^72^ have been developed to address these issues, each of them presents its own set of trade-offs. For example, MUV and DEKOIS 2.0 cover a narrower range of protein targets, and LIT-PCBA, which is based on experimental high-throughput screening data, can introduce artifacts such as assay interference and experimental false positives or negatives. Despite its imperfections, DUD-E remains a highly practical choice and serves as a de facto gold standard in the field. Utilizing DUD-E ensures that our performance metrics can be directly compared and contextualized within the vast existing literature on virtual screening methodologies, a critical benchmarking aspect that would be lost if every study adopted a completely different data set.

The results of our study intuitively rank BFE predictors according to their computational costs. The alchemical ABFE method demonstrated superior performance in both quantitative predictions and its ability to distinguish docked binders from nonbinders. One of the least expected findings was the reversal of roles between the end-state MD-based methods and ML models. While the end-state methods showed no correlation between calculated and experimental BFE values on the PDBbind data set, they performed relatively well in recognizing actives in the DUD-E data set. In contrast, the ML models outperformed the end-state methods on the PDBbind benchmark, likely because this subset overlaps with their training data. Interestingly, two ML models, Yuel and TopologyNet, performed worse on our PDBbind benchmark than on the broader PDBbind data set they were trained on, which may be attributed to overfitting to noise caused by the inclusion of noisy IC50 data as a proxy for binding affinity. An exception to the typical ML model performance pattern was GNINA. Unlike most ML models, GNINA behaved more like end-state physics-based methods—it underperformed on quantitative PDBbind data but clearly outperformed other ML methods, suggesting a higher degree of generalization. While most ML models performed poorly on the DUD-E data set, producing hit lists no better than random, GNINA’s performance was comparable to that of MM/GBSA. Overall, the benchmarked ML models consistently underperform on compounds that fall outside their training domain. Like many others,^34^ these models were trained on the PDBbind data set, which comprises a few thousand protein–ligand complexes with experimentally confirmed binding. Because they were never exposed to true nonbinders during training, it is unsurprising that they fail to distinguish binders from decoys in the DUD-E data set. The issue of domain applicability has been largely overlooked in previous studies, with only a recent effort^73^ explicitly investigating performance on out-of-distribution (OOD) compounds. However, even that study remains limited to the PDBbind data set and does not fully address the challenge of generalizing to true nonbinders.

In addition to overall performance metrics, we analyzed whether the benchmarked methods exhibit target-specific biases across the nine targets of the DUD-E benchmark (see Supplementary Tables S2, S3). The analysis revealed very weak correlations among the different methods, suggesting that their failure points are largely method-specific. For example, the end-state physics-based methods and Boltz-2 struggled on target HMDH, yielding numbers of true positives (NTP) at or below random levels (≤4), while several older ML models, such as Yuel and OnionNet-2, achieved true enrichment for this target (see Supplementary Table S3). In contrast, target GRIK1 proved highly challenging for the majority of the older ML methods, which failed to exceed random baseline performance, but was scored successfully by Boltz-2 and the alchemical method. Furthermore, target PGH2 presented a significant challenge for almost all ML methods, including Boltz-2, with only GNINA and RF-ScoreVS achieving meaningful enrichment. The alchemical binding free energy calculation was the only approach that maintained consistently high performance across the entire target panel (see Supplementary Table S2).

Finally, we also had a chance to run a small-scale analysis of how the reliance on docking poses as starting ligand geometries for alchemical simulations impacts the VS outcomes. While the use of docking is unavoidable, its negative impact on the overall true positive rate appears to be minimal. In a subset of 55 active ligands from the DUD-E benchmark, for which experimental X-ray structures are available, approximately 80% of the docking poses closely matched the native geometries—a high success rate that is well corroborated by extensive community docking benchmarks.^74,75^ Of the 12 divergent poses, four resulted in weaker predicted affinities, representing potential false negatives, but only one actually realized that risk. Therefore, on the basis of this limited assessment, one might expect to lose 2 to 5% of active compounds (if any) as false negatives in a typical screening campaign due to incorrect docking poses.

Beyond predictive accuracy, the practical utility of any VS triage method is heavily dictated by its computational cost and the availability of computing infrastructure. Comparing these costs directly is often challenging because calculation time is only one dimension of the overall expense, with hardware requirements and parallelization capacity playing equally critical roles. In our study, the evaluated methods generally fall into three distinct tiers of computational throughput: at the most resource-intensive end are the physics-based methods, which operate on the scale of hours per ligand. A full alchemical calculation requires approximately 2 h on a dedicated high-performance GPU on our local Hellbender cluster, and since GPU availability is typically limited, running multiple concurrent simulations is difficult. While MM/PBSA and MM/GBSA also require about 2 h per BFE calculation, they consume roughly 50 times fewer computing resources than alchemical methods, allowing for extensive concurrent execution across available nodes. The second tier consists of docking/cofolding ML models, such as GNINA and Boltz-2, which process complexes on a minute scale. Finally, the most rapid tier encompasses the machine learning models, which require only a single feed-forward pass and just a fraction of a second per complex. Because these standard machine-learning calculations demand very modest hardware resources, they can be massively parallelized to achieve extremely high screening throughputs.

Given the outcomes of this study, the alchemical ABFE method can be confidently recommended for prioritizing compounds in virtual screening, as it demonstrated consistently high performance with true positive rates between 86% and 100% across all targets. However, its significant computational cost may render it inaccessible for processing large compound libraries. Among the other methods evaluated, the AI model Boltz-2 emerged as a highly efficient alternative, performing comparably to end-state physics-based methods but with excellent consistency across different targets and at a fraction of the computational cost. If end-state methods must be used, then there is a clear preference for MM/PBSA over MM/GBSA. MM/GBSA underperformed on four of nine targets, highlighting the highly variable performance that practitioners might encounter and raising concerns about its reliability. To navigate this variability and computational cost, we recommend a multistage workflow. Practitioners can use an efficient AI approach like Boltz-2 to prefilter large compound libraries (on the order of 10^5^ ligands), followed by rigorous alchemical ABFE calculations on a few hundred top-ranked hits to ensure the high-accuracy identification of active compounds.

We hope that this study encourages the practical application of MD-based methods in hit finding for challenging therapeutic targets in the near future. These methods are expected to become more accessible with advancements in computational power and the increasing availability of affordable cloud-based solutions. While ML models are not yet ready for widespread deployment, we believe that advancements in learning strategies, including training on data sets beyond PDBbind and novel approaches to integrating ligand-protein interactions into neural networks, will drive future progress. Moreover, the data generated in this study, including MD trajectories, may contribute to improving both the physics-based and ML methods. This work could also inspire the development of learning models specifically trained on MD-derived data to predict binding affinity.

## Methods

### PDBbind Benchmark Set

A set of 4,284 unique ligands, corresponding to the refined subset of PDBbind^23^ comprising 5,316 protein–ligand complexes, was evaluated using a series of filtering criteria. The data were downloaded from PDBbind v2020 on 10/26/2022. The filters included: 1) amended Lipinski’s rules^36^ (molecular weight between 250 and 600 Da, cLogP between 1 and 5, hydrogen bond donors ≤ 5, hydrogen bond acceptors ≤ 10, and rotatable bonds < 14); and 2) structural filters (no phosphorus atoms, not more than two ionizable groups, at least one ring, no long aliphatic chains, no large fused-ring systems, no potentially toxic or highly reactive groups). All filters were implemented in Python using the RDKit library.^76^ Additionally, only ligands with activity data expressed as *K*_*d*_/*K*_*i*_ and a relation of “=” were retained. Overall, the filters reduced the data set to 1,286 protein–ligand complexes. Corresponding protein structures were downloaded from the RCSB PDB,^77^ and ligands were separated from the protein structures. Automated protein preparation (see below) failed to process 147 PDB files during automated processing steps (such as Open Babel ligand format conversion, protein chain splitting, incomplete 3D structure, etc.), resulting in a set of 1,139 protein–ligand complexes. Further, 285 metalloproteins were removed due to poor coverage by the standard force field. Some systems failed due to MD runtime errors, such as range-checking violations or memory-related issues. This set was later further reduced to 632 complexes (or 606 unique ligands).

### DUD-E Benchmark Set

Ligand structures in SMILES format were downloaded from the DUD-E Website on 4/29/2024 (https://dude.docking.org/). For the decoy set, 200 compounds per target were randomly selected, and Glide docking calculations were performed in “flexible” mode. The top 20 compounds per target were then chosen based on their ranked Glide scores. Ligands for the active set were selected using the same procedure as for the decoys; 135 compounds (15 per target) were chosen based on their docking scores. X-ray structures were available for 55 out of the 135 actives and were used as starting coordinates for MD simulations.

### Data Preparation

#### Ligand Structures

The structures were extracted from the PDB files, assigned bond orders, and had hydrogen atoms added by a Python code featuring the RDKit library. The resulting structures were saved as an SD file.

### Protein Preparation

Workflow in Maestro, Schrödinger Suite v2022–4 ^78^ was used to process the protein structures. The workflow added hydrogen atoms, reassigned bond orders, and generated missing residues. The protein protonation states were assigned using PROPKA at pH 7.0.

### Molecular Dynamics Data

*Bash* and Python scripts were used to prepare the MD input files. Topology and parameter files were generated using the AMBER14SB force field^79^ for proteins and the generalized AMBER force field (GAFF)^80^ for ligands. Ligand and protein structures were combined into a single ligand-protein complex in the GRO format. Energy minimization and equilibration MD runs were performed in batch mode by using a *bash* script. The integrity of ligand-protein complexes during MD simulations was evaluated by monitoring root-mean-square deviations (RMSD). Next, MM/GBSA, MM/PBSA, and FEP calculations were conducted for complexes that passed the integrity checks. Ultimately, MM/GBSA, MM/PBSA, and FEP calculations were successfully completed for 632 complexes.

### Docking

We utilized AutoDock Vina 1.2.0 ^14^ for molecular docking studies. The target proteins were prepared as PDBQT files using AutoDock Tools version 1.5.6. The grid box was set to 40 × 40 × 40 points in the x, y, and z dimensions, with a grid spacing of 0.375 Å. The center of the grid box was positioned at the ligand in the respective X-ray complexes to generate grid definition files (GDF files). Initial ligand structures in SMILES format were converted to three-dimensional geometries in PDB format using Open Babel. Hydrogen atoms were added to the resulting structures and further saved into PDBQT files required for AutoDock docking using the prepare_ligand4.py script from MGLTools. The default exhaustiveness parameter value of 8 was used.

### Molecular Dynamics

Molecular dynamics (MD) simulations were performed using GROMACS v2022.2 software.^79^ The AMBER14SB force field^81^ was applied for protein energy calculations, while ligand parametrization utilized the GAFF force field.^80^ Restrained electrostatic potential (RESP) charge parameters were derived from ESP charges calculated at the HF/6-31G* level using Gaussian 16.^82^ Ligand topologies in GROMACS format were prepared with the ANTECHAMBER module of the AMBER18 program via the Python script **acpype.py**.^83^ Each ligand-protein complex was placed in a cubic simulation box with a minimum distance of 10 Å between the complex and the box boundaries. Simulations were carried out in explicit solvent using the TIP3P water model, neutralized with 0.15 M NaCl. The system underwent energy minimization using the steepest descent algorithm to resolve steric clashes and unfavorable atomic interactions, terminating when the maximum force reached 2.4 kcal/mol/nm. This was followed by 100 ps equilibration runs in the NVT and NPT ensembles at 298 K and 1 atm, applying heavy-atom restraints of 239 kcal/mol/Å^2^ to stabilize temperature and pressure. The production phase consisted of 5 ns MD simulations without restraints, employing a velocity-rescaling thermostat.^84^ A 2 fs time step was used, with a 12 Å cutoff for nonbonded interactions. Long-range electrostatic interactions were calculated using the Particle Mesh Ewald (PME) method.^85^

### MM/GBSA and MM/PBSA Calculations

Both MM/GBSA and MM/PBSA exploit the following equation for BFE with default parameter settings as described in ref 86

2 $$\Delta G_{bind}=\Delta E_{MM}+\Delta G_{polar}+\Delta G_{non‐polar}-T\Delta S$$

where Δ*E_MM_* is the system’s energy in the gas phase calculated by the molecular mechanics (MM) force field, Δ*G_polar_* is the polar solvation free energy calculated either by the Poisson–Boltzmann or Generalized Born model, Δ*G_non_*_–_ *_polar_* is the non-polar solvation energy estimated by the weighted solvent-accessible surface area (SASA), and *T*Δ*S* is the entropic contribution. Two alternative methods, *normal-mode analysis* ^50,51^ and *IE* ^52^ were used to calculate the entropic contribution. All calculations were performed using the gmx_MMPBSA v1.6.2 tool.^87,88^ The last 1 ns of 5 ns MD trajectories (1,000 snapshots) were used for the BFE calculations. The normal-mode analysis was limited to 20 frames due to its high computational cost. The IE calculations involved all 1,000 frames.

### Alchemical Free Energy Calculations

The core portion of our workflow was adapted from a GROMACS tutorial,^89^ further automated for high-throughput use, and tailored to the Hellbender cluster at the University of Missouri. Prior to simulations, a set of 25 λ-windows was generated for each of the two coupling routes: ligand coupling in water and ligand coupling to the protein in solvent. The λ-windows span the range from λ = 0.0 to 1.0, with increments of 0.1 up to λ = 0.8 and 0.05 from λ = 0.85 to 1.0. In the first 12 windows, the van der Waals (vdW) potential was gradually activated while keeping Coulombic interactions turned off. In the remaining windows, Coulombic interactions were progressively introduced. The Bennett Acceptance Ratio (BAR) method^45^ was used to calculate Δ*G* between states. Soft-core parameter settings were defined as sc–alpha = 0.5, sc–power = 1, and sc–sigma = 0.3 to prevent overlapping particles during the simulations.^90^ The values of Δ*H*/ λ were recorded at 100 fs intervals. The complete BFE thermodynamic cycle comprised 50 λ-windows, with MD simulations of 5 ns per window. Orientational restraints were implemented using the Boresch–Karplus algorithm.^91^

### Machine Learning BFE Predictors

#### KDEEP

Proteins were prepared by removing ligands and adding hydrogen atoms using the OpenBabel library; the processed structures were saved in the PDB format. Ligands were prepared by generating their 3D coordinates within a predefined box and adding hydrogen atoms; the processed ligand structures were saved in the SD format. The prepared input files were uploaded to OpenPlayMolecule.org, where KDEEP was run online (on 11/16/2024) to calculate BFE values.

#### OnionNet-2

The protein and ligand structures were prepared using the same procedure as that for KDEEP. The OnionNet-2 environment was set up by following the installation instructions provided in the OnionNet GitHub repository. The OnionNet-2 code was downloaded from github.com/zchwang/OnionNet-2 and run with the prepared input files (on 11/18/2024) to predict the BFE values.

#### TopologyNet

The protein and ligand structures were prepared using the same procedure as that for the above two methods. The TopologyNet environment was set up by following the installation instructions provided on the TopologyNet GitHub repository. Once the environment was configured, the prepared input files were used to run the TopologyNet code (on 11/20/2024) to predict the BFE values.

#### Yuel

Proteins were prepared as one-letter sequences in plain text format, ligands as SMILES strings, and both were saved in an Excel file. The environment for Yuel was set up by following the installation instructions. The code and pretrained model were downloaded from bitbucket.org/dokhlab/yuel/src/master and run on 11/22/2024 to calculate the BFE values.

#### GNINA

Proteins were prepared by removing the target chain using the OpenBabel library; the processed structures were saved in PDB format. The unchanged ligand structures were used to define the docking box coordinates; no further modifications were made to the ligands. The GNINA environment was set up using the Google Colab notebook provided at github.com/gnina/gnina. GNINA docking was run on 1/3/2025 to predict the BFE values by using its default scoring function.

### Hypergeometric Distributions

To evaluate the performance of the BFE predictor in virtual screening (VS) and to extrapolate its results to larger data sets, we used the hypergeometric distribution implemented in Python’s *scipy.stats.hypergeom* module. Two specific analyses were performed. First, the probability of observing exactly *k_obs_* true actives (*P*(*k_obs_*)) in the experimental data set was calculated using the probability mass function (PMF) of the hypergeometric distribution. The PMF is computed using *hypergeom*.*pmf*(*k*,*N*,*K*,*n),* where *N* is the total population size, *K* is the total number of true actives, *N* is the sample size, and *K* is the observed number of true actives. This calculation determined whether the observed number of true actives could reasonably occur by random chance, providing a quantitative assessment of enrichment. Second, to extrapolate the expected number of true actives (*k*_2_) in a larger data set, the cumulative probability *P*(*k*≥*k*_2_) observed in the experimental setup was matched to the corresponding cumulative probability in the scaled-up scenario. The cumulative probability *P*(*k*≥*k*_2_) for a given data set is computed using 1–*hypergeom*.*cdf*(*k*–1,*N*,*k*,*n*), where *hypergeom*.*cdf* represents the cumulative distribution function of the hypergeometric distribution. For the scaled-up data set (the real-world VS scenario) with parameters *k*_2_,*N*_2_,*K*_2_,*n*_2_, we iteratively solved for *k*_2_ such that the cumulative probability *P*(*k*≥*k*_2_) closely matched *P*(*k*≥*k_obs_*) from the experimental setup. The smallest *k*_2_ satisfying this condition was identified.

## Supplementary Material

















## Data Availability

All input and configuration files, command-line scripts, and Python code for running physics-based and ML methods can be found at https://github.com/kireevlab/Last-mile-physics-based-AI-for-VS.

## Notes

Supplementary Figures S1–S5 and Supplementary Tables S1–S4 (PDF)
Supplementary Data Set 1: Protein structures from the PDBbind data set in compressed PDB format (ZIP)
Supplementary Data Set 2: A table of unique ligands from the PDBbind data set; contains ID, SMILES, and rule-of-5 properties (XLSX)
Supplementary Data Set 3: A table of unique proteins from the PDBbind data set; contains UniProt ID, ligand count, and protein name (XLSX)
Supplementary Data Set 4: Structures of protein–ligand complexes from the DUD-E data set in compressed PDB format (ZIP)
Supplementary Data Set 5: A table of unique ligands from the DUD-E data set; contains ID, SMILES, Lipinski rule-of-5 properties, target, and activity status (XLSX)
Supplementary Data Set 6: A table of experimental and calculated BFE values for the PDBbind data set (XLSX)
Supplementary Data Set 7: A table of experimental and calculated BFE values for the DUD-E data set (XLSX)
