# Supplementary material for: The Last Mile Problem: A Critical Assessment of Physics-Based and AI Tools for Small Molecule Binding Prediction in Virtual Screening
Source: J Chem Inf Model. 2026 May 7;66(10):5964–77. doi: 10.1021/acs.jcim.6c00942 (PMC13213910; doi:10.1021/acs.jcim.6c00942)
Supplement: Supplementary file 1 [file ci6c00942_si_001.pdf]

The Last Mile Problem: A Critical Assessment of Physics-based and AI Tools for Small Molecule Binding Prediction in Virtual Screening

Xiaowen Wang, Hamza Hentabli, Akhila Mettu, Shubha Gautam, Dmitri Kireev*

Department of Chemistry, College of Arts and Sciences, University of Missouri – Columbia

*E–mail address for correspondence: dmitri.kireev@missouri.edu

Supplementary figures

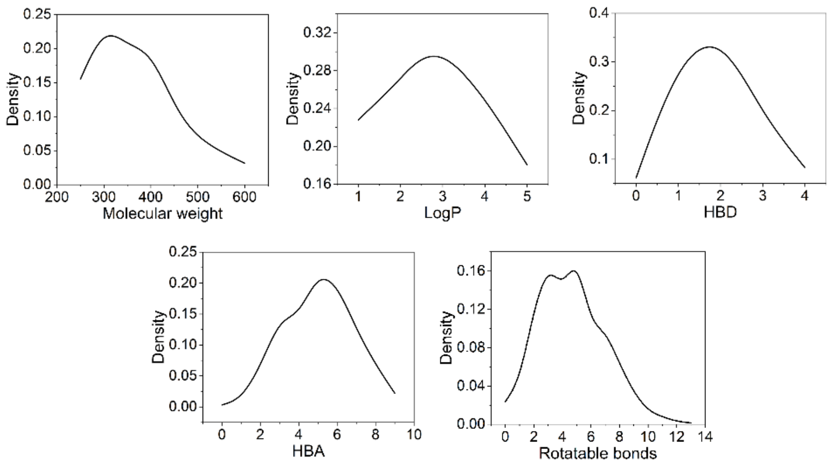

Supplementary Figure S1. *Probability density functions of the Lipinski properties(HBD: Hydrogen bond Donors, HBA: Hydrogen bond acceptors) and rotatable bonds for the PDBbind set of 606 ligands.*

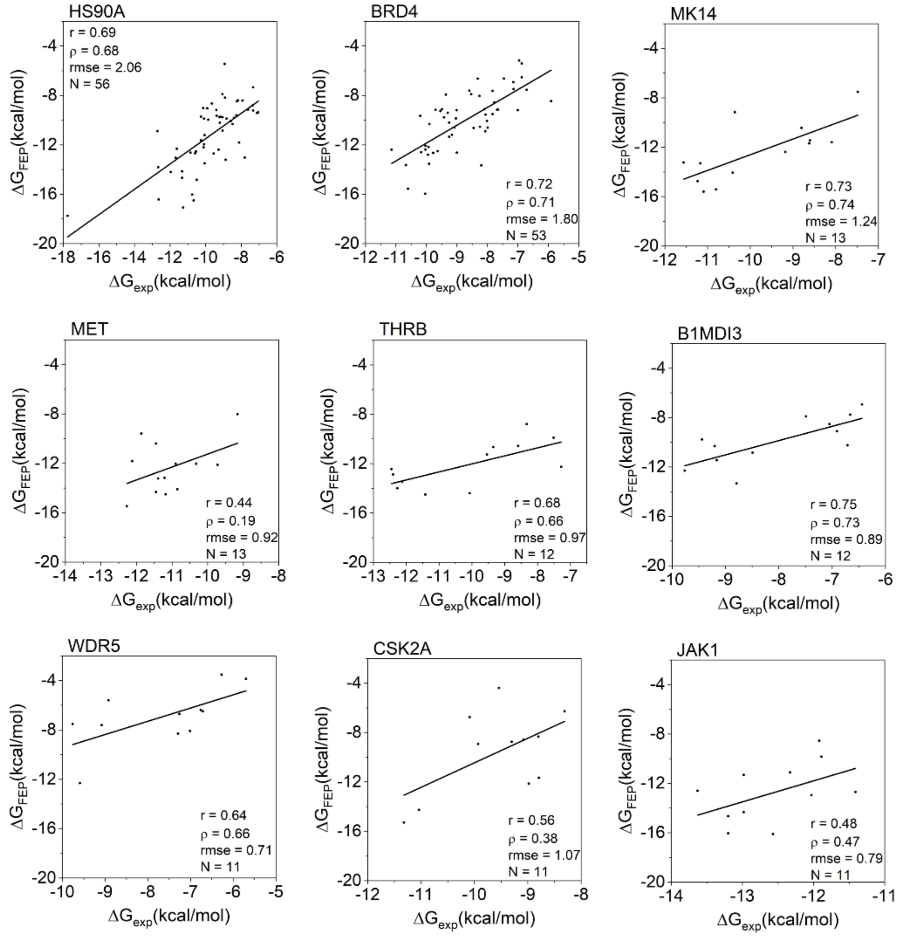

Supplementary Figure S2. *Intra-target correlations between experimental BFE (ΔG_{exp}) and the alchemical ABFE.*

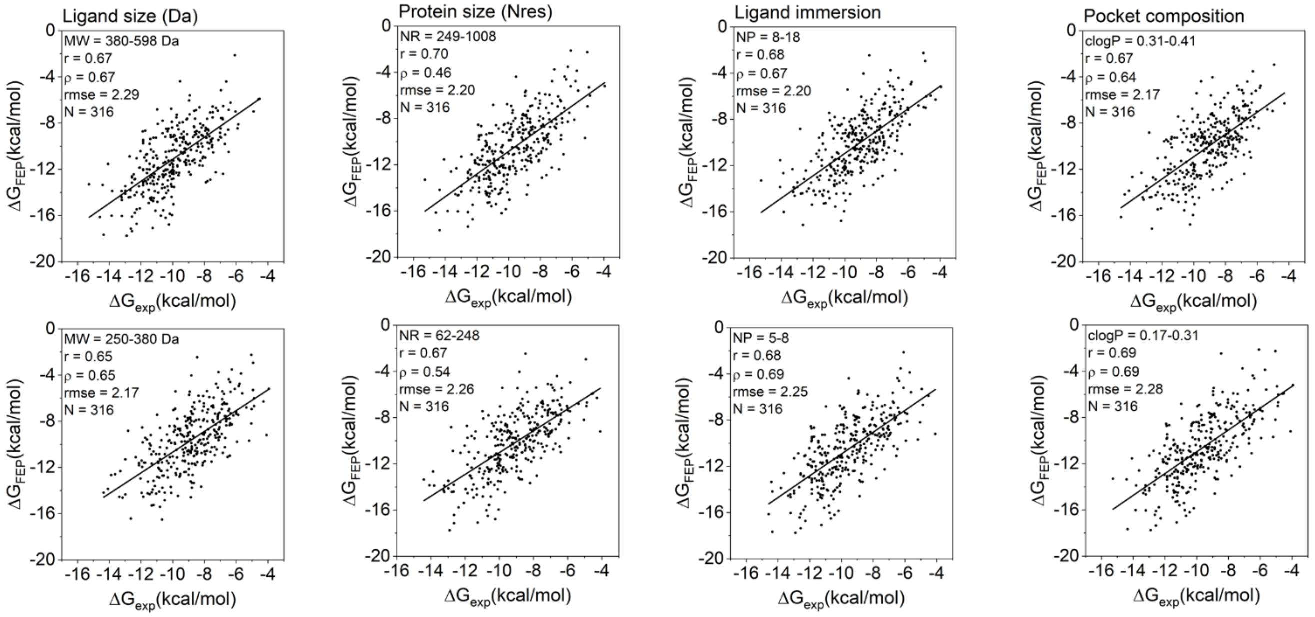

Supplementary Figure S3. *Correlations between experimental BFE (ΔG_{exp}) and the alchemical ABFE for complexes with properties above or below the medians of ligand size (expressed as molecular weight (Da)), protein size (expressed as the number of residues), ligand immersion (expressed as the mean number of nearby protein numbers per ligand atom), and pocket composition (expressed as mean clogP of the binding-site residues).*

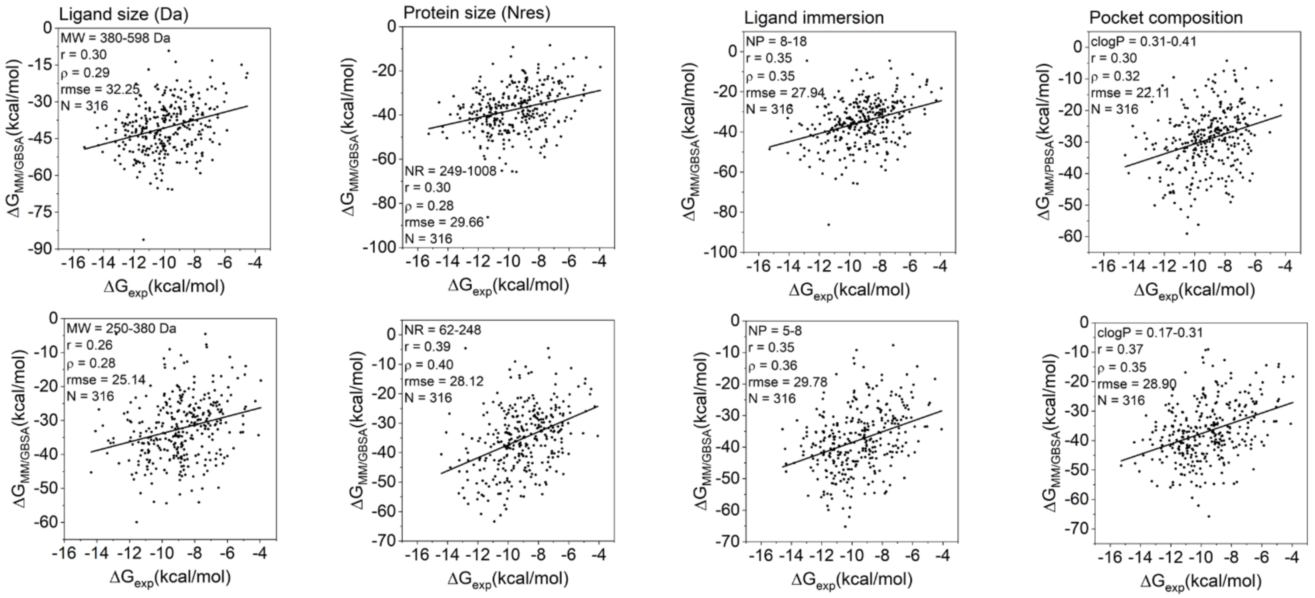

Supplementary Figure S4. *Correlations between experimental BFE (ΔG_{exp}) and the MM/GBSA for complexes with properties above or below the medians of ligand size (expressed as molecular weight (Da)), protein size (expressed as the number of residues), ligand immersion (expressed as the mean number of nearby protein numbers per ligand atom), and pocket composition (expressed as mean clogP of the binding-site residues).*

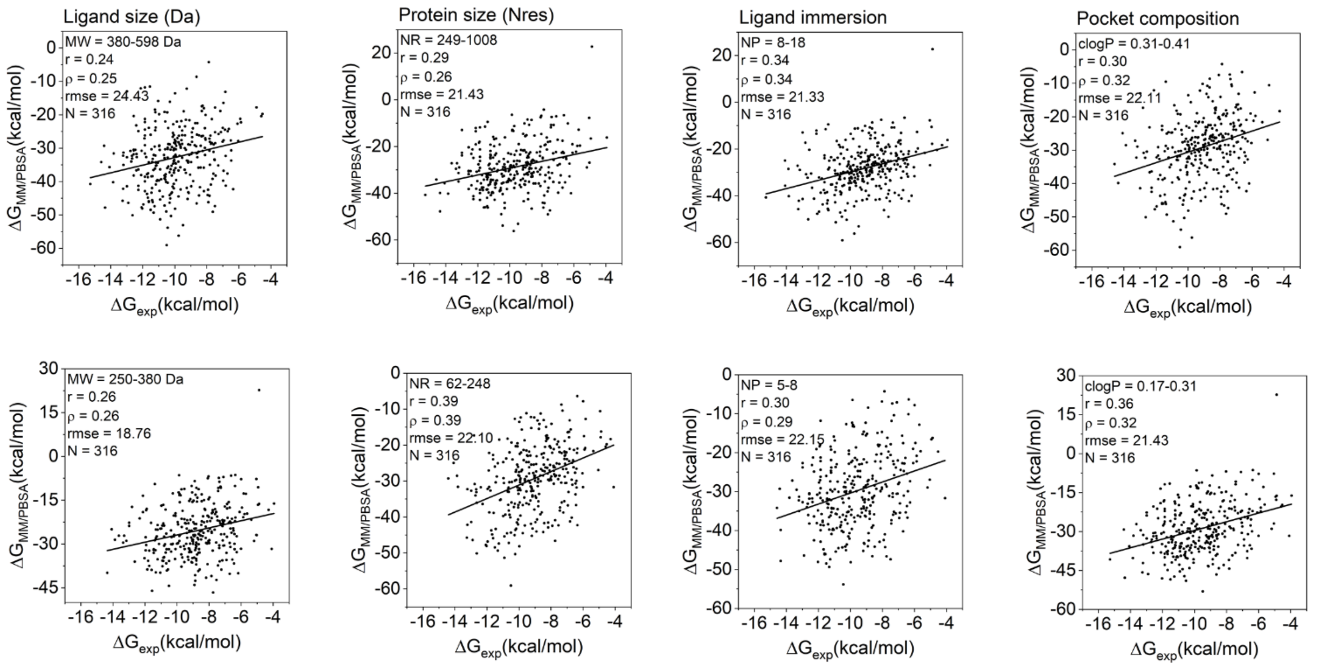

Supplementary Figure S5. *Correlations between experimental BFE (ΔG_{exp}) and the MM/PBSA for complexes with properties above or below the medians of ligand size (expressed as molecular weight (Da)), protein size (expressed as the number of residues), ligand immersion (expressed as the mean number of nearby protein numbers per ligand atom), and pocket composition (expressed as mean clogP of the binding-site residues)*

Supplementary Tables

Supplementary Table S1. Protein superfamilies and their corresponding protein counts in the PDBbind dataset.

SUPFAM_ID	COUNT	SUPFAM_NAME
SSF56112	31	Protein kinase-like (PK-like)
SSF50494	18	Trypsin-like serine proteases
SSF48508	12	Nuclear receptor ligand-binding domain
SSF47370	11	Bromodomain
SSF48726	11	Immunoglobulin
SSF46919	11	N-terminal Zn binding domain of HIV integrase
SSF50630	9	Acid proteases
SSF53474	8	alpha/beta-Hydrolases
SSF47473	7	EF-hand
SSF50814	7	Lipocalins
SSF52540	6	P-loop containing nucleoside triphosphate hydrolases
SSF50044	6	SH3-domain
SSF47090	6	PGBD-like
SSF51069	5	Carbonic anhydrase
SSF53822	5	Periplasmic binding protein-like I
SSF49899	5	Concanavalin A-like lectins/glucanases
SSF51735	5	NAD(P)-binding Rossmann-fold domains
SSF52768	5	Arginase/deacetylase
SSF48371	5	ARM repeat
SSF54427	4	NTF2-like
SSF11094	4	N/A
SSF56601	4	beta-lactamase/transpeptidase-like
SSF54211	4	Ribosomal protein S5 domain 2-like
SSF56281	4	Metallo-hydrolase/oxidoreductase
SSF46689	4	Homeodomain-like
SSF14292	3	N/A
SSF52799	3	(Phosphotyrosine protein) phosphatases II
SSF47769	3	SAM/Pointed domain
SSF54001	3	Cysteine proteinases
SSF63712	3	Nicotinic receptor ligand binding domain-like
SSF47031	3	Second domain of FERM
SSF56420	3	Peptide deformylase
SSF53167	3	Purine and uridine phosphorylases
SSF53448	2	Nucleotide-diphospho-sugar transferases
SSF75217	2	alpha/beta knot

SSF48619	2	Phospholipase A2, PLA2
SSF51445	2	(Trans)glycosidases
SSF51161	2	Trimeric LpxA-like enzymes
SSF55550	2	SH2 domain
SSF56854	2	Bcl-2 inhibitors of programmed cell death
SSF48452	2	TPR-like
SSF50891	2	Cyclophilin-like
SSF47986	2	DEATH domain
SSF54236	2	Ubiquitin-like
SSF16045	2	N/A
SSF54593	2	Glyoxalase/Bleomycin resistance protein/Dihydroxybiphenyl dioxygenase
SSF53597	2	Dihydrofolate reductase-like
SSF56574	2	Serpins
SSF46785	2	"Winged helix" DNA-binding domain
SSF52949	2	Macro domain-like
SSF55831	2	Thymidylate synthase/dCMP hydroxymethylase
SSF49472	2	Transthyretin (synonym: prealbumin)
SSF47836	2	Retroviral matrix proteins
SSF52490	1	Tubulin nucleotide-binding domain-like
SSF49842	1	TNF-like
SSF52440	1	PreATP-grasp domain
SSF54637	1	Thioesterase/thiol ester dehydrase-isomerase
SSF52304	1	Type II 3-dehydroquinate dehydratase
SSF11783	1	N/A
SSF50876	1	Avidin/streptavidin
SSF56219	1	DNase I-like
SSF50405	1	Actin-crosslinking proteins
SSF55874	1	ATPase domain of HSP90 chaperone/DNA topoisomerase II/histidine kinase
SSF10324	1	N/A
SSF53697	1	SIS domain
SSF55035	1	NAD-binding domain of HMG-CoA reductase
SSF47719	1	p53 tetramerization domain
SSF50370	1	Ricin B-like lectins
SSF74650	1	Galactose mutarotase-like
SSF47095	1	HMG-box
SSF50939	1	Sialidases
SSF89807	1	Dodecin-like
SSF11728	1	N/A
SSF54928	1	RNA-binding domain, RBD
SSF50353	1	Cytokine

SSF11133	1	N/A
SSF52200	1	Toll/Interleukin receptor TIR domain
SSF47943	1	Retrovirus capsid protein, N-terminal core domain
SSF48403	1	Ankyrin repeat
SSF51283	1	dUTPase-like
SSF56784	1	HAD-like
SSF57903	1	FYVE/PHD zinc finger
SSF49299	1	PKD domain
SSF54736	1	ClpS-like
SSF47565	1	Insect pheromone/odorant-binding proteins
SSF47040	1	Kix domain of CBP (creb binding protein)
SSF56502	1	gp120 core
SSF49401	1	Bacterial adhesins
SSF53098	1	Ribonuclease H-like
SSF51246	1	Rudiment single hybrid motif
SSF14012	1	N/A
SSF54695	1	POZ domain
SSF55811	1	Nudix
SSF52833	1	Thioredoxin-like
SSF48552	1	Serum albumin-like
SSF52335	1	Methylglyoxal synthase-like
SSF55331	1	Tautomerase/MIF
SSF46774	1	ARID-like
SSF55021	1	ACT-like
SSF51713	1	tRNA-guanine transglycosylase
SSF48097	1	Regulator of G-protein signaling, RGS
SSF50249	1	Nucleic acid-binding proteins
SSF47459	1	HLH, helix-loop-helix DNA-binding domain
SSF53300	1	vWA-like
SSF47055	1	TAF(II)230 TBP-binding fragment
SSF51717	1	Dihydropteroate synthetase-like
SSF51690	1	Nicotinate/Quinolinate PRTase C-terminal domain-like
SSF47175	1	Cytochromes
SSF55718	1	SCP-like
SSF47592	1	SWIB/MDM2 domain
SSF51230	1	Single hybrid motif
SSF53901	1	Thiolase-like
SSF10191	1	N/A
SSF47616	1	GST C-terminal domain-like
SSF51366	1	Ribulose-phoshate binding barrel

SSF75304	1	Amidase signature (AS) enzymes
SSF52129	1	Caspase-like
SSF57552	1	Blood coagulation inhibitor (disintegrin)
SSF53748	1	Phosphoglycerate kinase
SSF50978	1	WD40 repeat-like
SSF47266	1	4-helical cytokines
SSF51556	1	Metallo-dependent hydrolases
SSF55186	1	ThrRS/AlaRS common domain
SSF53681	1	Aspartate/glutamate racemase

Supplementary Table S2. Pearson's correlation coefficient (*R*) and root mean square error (rmse) for intra-target correlations on the PDBbind benchmark set.

	Alchemical		MM/GBSA		MM/PBSA		KDEEP		OnionNet-2		TopologyNet		Yuel		GNINA	
Target	R	rmse	R	rmse	R	rmse	R	rmse	R	rmse	R	rmse	R	rmse	R	rmse
HS90A	0.69	2.1	0.35	28.1	0.38	22.2	0.92	0.56	0.83	0.87	0.29	1.49	0.42	4.00	0.13	2.01
BRD4	0.72	1.8	0.53	18.5	0.59	15.8	0.96	0.33	0.80	0.82	0.60	0.94	0.43	3.98	0.33	1.24
MK14	0.73	1.2	0.45	14.1	0.61	9.9	0.98	0.28	0.94	0.56	0.28	1.29	0.65	1.05	0.66	1.07
THRB	0.68	1.0	0.21	11.8	0.14	8.4	0.99	0.16	0.69	1.47	0.19	1.90	0.22	3.62	0.39	1.91
MET	0.44	0.9	0.02	13.17	0.03	9.47	0.29	1.23	0.71	0.61	0.08	0.99	0.60	3.79	0.11	1.24
B1MDI3	0.75	0.9	0.20	12.4	-0.14	9.69	0.66	0.97	0.61	1.01	0.50	1.04	0.11	1.57	0.48	1.05
WDR5	0.64	0.7	0.71	8.1	0.50	5.79	0.96	0.44	0.32	1.88	0.24	1.69	0.89	1.23	0.83	0.91
CSK2A	0.56	1.1	0.49	8.22	0.20	5.64	0.95	0.28	0.83	0.66	0.20	2.01	0.50	1.00	-0.03	1.94
JAK1	0.48	0.8	0.10	8.77	0.12	6.00	0.31	1.04	-0.20	1.41	0.54	2.75	0.21	5.12	0.32	3.43

Supplementary Table S3. Numbers of True Positives (NTP), True Positive Rates (TPR), Probabilities to observe the TPR in a random selection (P(k)), and projected NTP in a practical VS setting (k_{VS}) for MM/GBSA, MM/PBSA, and alchemical ABFE.

	**MM/GBSA**				**MM/PBSA**				**Alchemical**			
**Target**	**NTP**	**TPR (%)**	***P(k)***	***k*$_{VS}$**	**NTP**	**TPR (%)**	***P(k)***	***k*$_{VS}$**	**NTP**	**TPR (%)**	***P(k)***	***k*$_{VS}$**
**ACE**	4	57	2.3E-01	2	5	71	8.5E-02	3	7	100	9.6E-04	5
**ADRB1**	5	71	8.5E-02	3	6	86	1.5E-02	4	7	100	1.2E-03	5
**FAK1**	7	100	9.6E-04	5	7	100	9.6E-04	5	7	100	9.6E-04	5
**GRIK1**	5	71	8.5E-02	3	5	71	8.5E-02	3	7	100	9.6E-04	5
**HMDH**	1	14	1.9E-01	0	2	29	3.4E-01	1	6	86	1.5E-02	4
**MCR**	6	86	1.5E-02	4	6	86	1.5E-02	4	6	86	1.5E-02	4
**PGH2**	4	57	2.8E-01	2	7	100	1.9E-03	5	5	71	8.5E-02	3
**PRGR**	7	100	9.6E-04	5	7	100	9.6E-04	5	6	86	1.8E-02	4
**TRYB1**	7	100	9.6E-04	5	7	100	9.6E-04	5	7	100	2.4E-03	5
**Total**	46	73	1.8E-07	8	52	83	2.5E-12	10	58	92	1.7E-20	10

Supplementary Table S4. Numbers of True Positives (NTP), True Positive Rates (TPR), Probabilities to observe the TPR in a random selection (P(k)), and projected NTP in a practical VS setting (K_{VS}) for the ML-based BFE predictors.

	KDEEP				Yuel				OnionNet-2				TopologyNet			
**Target**	**NTP**	**TPR**	***P(k)***	k_{VS}	**NTP**	**TPR**	***P(k)***	k_{VS}	**NTP**	**TPR**	***P(k)***	k_{VS}	**NTP**	**TPR**	***P(k)***	k_{VS}
**ACE**	2	29	2.4E-01	0	3	43	3.3E-01	1	2	29	2.4E-01	0	5	71	8.5E-02	3
**ADRB1**	5	71	8.5E-02	3	1	14	8.6E-02	0	2	29	2.4E-01	0	3	43	3.3E-01	1
**FAK1**	4	57	2.3E-01	2	6	86	1.5E-02	4	1	14	8.6E-02	0	3	43	3.3E-01	1
**GRIK1**	2	28	2.4E-01	0	2	29	2.4E-01	0	2	29	2.4E-01	0	1	14	8.6E-02	0
**HMDH**	5	71	8.5E-02	3	6	86	1.5E-02	4	6	86	1.5E-02	4	5	71	8.5E-02	3
**MCR**	2	29	2.4E-01	0	5	71	8.5E-02	3	6	86	1.5E-02	4	0	0	1.2E-02	0
**PGH2**	3	43	3.3E-01	1	3	43	3.3E-01	1	2	29	2.4E-01	0	1	14	8.6E-02	0
**PRGR**	5	71	8.5E-02	3	3	43	3.3E-01	1	3	43	3.3E-01	1	5	71	8.5E-02	3
**TRYB1**	5	71	8.5E-02	3	2	29	2.4E-01	0	6	86	1.5E-02	4	4	57	2.3E-01	2
**Total**	33	52	8.6E-02	3	31	49	2.1E-01	2	30	48	7.8E-02	2	30	48	3.0E-01	2

	GNINA				Boltz-2				RF-ScoreVS			
**Target**	**NTP**	**TPR**	***P(k)***	k_{VS}	**NTP**	**TPR**	***P(k)***	k_{VS}	**NTP**	**TPR**	***P(k)***	k_{VS}
**ACE**	4	57	2.3E-01	2	7	100	9.6E-04	5	7	100	9.6E-04	5
**ADRB1**	4	57	2.3E-01	2	7	100	9.6E-04	5	6	86	1.5E-02	4
**FAK1**	7	100	9.6E-04	5	6	86	1.5E-02	4	7	100	9.6E-04	5
**GRIK1**	2	29	2.4E-01	0	6	86	1.5E-02	4	3	43	3.3E-01	1
**HMDH**	6	86	1.5E-02	4	3	43	3.3E-01	1	3	43	3.3E-01	1
**MCR**	5	71	8.5E-02	3	7	100	9.6E-04	5	7	100	9.6E-04	5
**PGH2**	5	71	8.5E-02	3	2	28	2.4E-01	0	5	71	8.5E-02	3
**PRGR**	2	29	2.4E-01	0	6	86	1.5E-02	4	7	100	9.6E-04	5
**TRYB1**	3	43	3.3E-01	1	7	100	9.6E-04	5	5	71	8.5E-02	3
**Total**	38	60	2.7E-03	5	51	81	2.1E-11	10	50	79	1.6E-10	10
